# Initiating the antiretroviral therapy in treatment-naïve patients

**DOI:** 10.1186/1471-2334-14-S7-P85

**Published:** 2014-10-15

**Authors:** Elisabeta Benea, Raluca Jipa, Cozmina Andrei, Mariana Mărdărescu, Eliza Manea, Iulia Niculescu, Șerban Benea, Ruxandra Moroti, Adriana Hristea

**Affiliations:** 1 National Institute for Infectious Diseases "Prof. Dr. Matei Balş", Bucharest, Romania; 2 Carol Davila University of Medicine and Pharmacy, Bucharest, Romania

## Background

The national guideline extends the recommendation for initiating antiretroviral therapy in HIV infected persons, regardless of baseline CD4 since 2013 but the choice of treatment regimens in our guide is determined by the CD4 levels. Objectives: analyzing ART regimens prescribed in newly HIV-infected patients, identifying the factors that influenced regimens choice and analyzing viro-immunological evolution under therapy.

## Methods

Retrospective study of adult patients with confirmed HIV infection who initiated antiretroviral treatment in a hospital for infectious diseases in Bucharest, between January 2012 and December 2013.

## Results

Of the 499 patients confirmed with HIV infection, 243 (48.70%) have initiated antiretroviral therapy. Comparative analysis shows that treated patients have a higher median age (33.39 vs. 30.89 years; p=0.002), with a significantly higher proportion of women (82/243 vs. 55/256; p=0.002) and a percentage of IV drug users significantly lower (45/243 vs. 190/256; p=0.00001).

Schemes with 2 NRTIs+PIs were preferred (137 patients; 56.38%) followed by those with 2 NRTIs+NNRTIs (80 patients; 32.92%), 2 NRTIs+IIs (15 patients; 6.17%) and other schemes (11 patients; 6.17%). The choice of the ARV regimen was not influenced by age, gender, route of transmission and RNA-HIV1 at baseline.

The proportion of patients with CD4<350 cells/cmm at baseline was significantly higher in those treated with 2NRTIs+PIs, compared to those treated with 2NRTIs+NNRTIs (106/137 vs. 51/80; p=0.004). Patients co-infected with HIV/HCV and HIV/HBV were most commonly treated with IIs (8/15 vs. 12/80; p=0.009) and PIs (47/137 vs. 12/80; p=0.001) than with NNRTIs. Patients co-infected with *M. tuberculosis* most frequently received regimens with NNRTIs (17/80 vs. 2/137; p=0.000001), IIs (4/15 vs. 17/80; p=0.0008) and other schemes (3/11 vs. 2/137; p=0.002) than with PIs.

There were no statistically significant differences in the CD4 cell count increase at 6 and 12 months of therapy, or in the percentage of patients with undetectable RNA-HIV1 after 6 months of treatment. The percentage of patients with VL undetectable at 6 months was 51.06% for those treated with NNRTIs, 34.29% for those treated with PIs, 30.7% for those treated with IIs and 20% for those treated with other regimens. These values reflect the important issues of adherence to ARV therapy seen in our patients.

## Conclusion

In our patients a CD4 cell count below 350 cells/cmm and co-infections influence the choice of the ARV regimen. Inadequate adherence is responsible for the small percentage of patients with VL undetectable after 6 months of treatment.

